# Sensor-Informed Motion-Continuity Control of Shared-Return Electro-Hydraulic Actuator Networks Under Neighboring-Branch Disturbances

**DOI:** 10.3390/s26154739

**Published:** 2026-07-26

**Authors:** Tiangu Wu, Lijuan Zhao, Guocong Lin, Shutian Gong

**Affiliations:** 1 School of Mechanical Engineering, Liaoning Technical University, Fuxin 123000, China; 2 Liaoning Provincial Technical Innovation Center for Hydraulic Transmission and Control, Fuxin 123000, China

**Keywords:** sensor-informed control, pressure sensing, displacement sensing, shared-return hydraulic actuator networks, electro-hydraulic control, motion continuity, predictive control, soft actor–critic

## Abstract

This study focuses on the development of a sensor-informed motion-continuity control method for shared-return electro-hydraulic actuator networks subject to neighboring-branch disturbances. The objective is to reduce the local velocity fluctuations induced by return-line pressure transients while retaining explicit hydraulic and valve constraints. A control-oriented shared-return disturbance model is established to map neighboring-valve action, T-port replenishment, accumulator buffering, common return-line pressure, net driving pressure difference, and local actuator motion. On this basis, sensor-derived motion and pressure states together with neighboring-action prior information are used to reconstruct the objective of a constrained predictive controller according to the disturbance stage. Soft Actor–Critic is restricted to bounded objective-weight inference, whereas the valve command remains generated by locally linearized receding-horizon optimization; bounded mapping, smoothing update, and soft pressure constraints preserve positive weighting matrices and online quadratic programming solvability. Co-simulation, simulation-based ablation and baseline comparisons, timing evaluation, and scaled dual-branch experiments show that the proposed framework improves motion continuity, reduces disturbance-induced pressure-difference excursions, maintains smoother valve execution, and completes each tested online update within the sampling period. These findings support feasibility-preserving sensor-driven objective reconstruction under the investigated shared-return disturbance scenarios.

## 1. Introduction

Networked hydraulic actuator systems are widely used in heavy-duty equipment, mining machinery, construction machinery, and other engineering systems requiring high load capacity and coordinated motion. In these systems, multiple actuator units are usually supplied by common hydraulic sources and connected to shared supply–return manifolds rather than operating as fully independent subsystems. Consequently, the motion of one actuator is not only determined by its local valve command and mechanical load, but is also affected by pressure and flow variations induced by neighboring actuator actions through the shared hydraulic network. In coordinated conveyor pushing of hydraulic support groups, sequential advancing, valve switching, and start–stop operations of adjacent supports may lead to rapid variations in local flow demand and return-line pressure, which further influence the net driving pressure difference and velocity response of the controlled actuator [1,2,3,4]. These interactions form a networked nonlinear hydraulic dynamic system with shared-resource coupling.

The nonlinear dynamic behavior of such systems is mainly caused by three coupled mechanisms. First, the pressure–flow relation of hydraulic valves and pipelines is inherently nonlinear, especially under valve switching and changing pressure-drop conditions. Second, the compressibility of the working fluid and the time-varying chamber volume of the hydraulic cylinder introduce strong pressure–motion coupling. Third, the shared return line provides a propagation path through which neighboring-unit disturbances can alter the discharge boundary of the local actuator. When the neighboring actuator suddenly changes its valve opening or stops moving, the resulting return-side transient may cause a short-duration pressure drop or pressure fluctuation in the common return manifold. This pressure variation changes the rod-chamber discharge condition of the controlled actuator, thereby modifying its net driving pressure difference and generating velocity overshoot or motion discontinuity. Although hydraulic support sensing, intelligent decision-making, positioning control, and supply-pressure stabilization have been extensively studied [1,2,3,4,5], the dynamic chain from neighboring-valve action to shared return-line pressure propagation, local replenishment activation, pressure-difference variation, and actuator velocity response has not yet been sufficiently modeled from a nonlinear dynamic perspective.

Hydraulic pipeline transients and accumulator buffering provide important theoretical references for understanding shared-line disturbances. Existing studies on water hammer, hydraulic pipeline vibration, and high-flow directional-valve transients have shown that pipeline impedance, fluid inertia, valve-switching rate, and accumulator configuration can significantly affect pressure peaks, pressure recovery, and vibration propagation in hydraulic systems [6,7,8]. However, most of these studies focus on single-pipeline transients, local valve-induced water hammer, or component-level buffering effects. For networked hydraulic actuator arrays, the relevant mechanisms include pressure-fluctuation propagation through the shared return manifold, pressure-difference-driven activation of the neighboring T-port replenishment branch, accumulator buffering, and the resulting influence on local actuator motion continuity. These mechanisms are represented by a compact nonlinear model for mechanism analysis and controller design.

Electro-hydraulic actuators are difficult to regulate because valve-flow nonlinearity, fluid compressibility, friction, load variation, uncertainty, saturation, and limited disturbance measurements act simultaneously. Existing output-feedback MPC, force control, multi-cylinder synchronization, adaptive or neural control, disturbance compensation, and observer-based methods use local measurements to regulate isolated actuators and local mechanisms [9,10,11,12,13,14,15,16,17,18,19]. In a shared-return network, however, displacement, velocity, and manifold-pressure signals also encode disturbances transmitted from neighboring branches. How to use these signals and the neighboring-action prior to reconstruct a control objective across disturbance stages remains insufficiently addressed.

Reinforcement learning offers state-dependent adaptation, and SAC is suitable for continuous actions because of its entropy-regularized off-policy formulation [20,21]. Related studies have applied RL to hydraulic-servo control, hydraulic-support alignment, and model-based hydraulic control [22,23,24]. Direct RL valve policies, however, provide limited explicit enforcement of pressure and input constraints. The present architecture therefore assigns bounded objective-weight inference to SAC and retains constrained valve-command optimization in the predictive layer [25,26,27,28].

Mechanical–hydraulic–control co-simulation provides a validation environment for the nonlinear dynamic model and learning-enhanced predictive controller. The coupled environment represents mechanical load transfer, hydraulic pressure–flow transients, and online controller execution [29,30].

Taken together, prior studies separately address pipeline transients, local actuator regulation, learning-based adaptation, or co-simulation, but not the complete neighboring-action–return-manifold–local-motion chain with state-dependent constrained objective reconstruction.

The proposed controller uses sensor-derived motion and pressure states, neighboring-action information, and the previous input to reconstruct three bounded DMPC weights; the constrained lower layer alone generates the valve command [22,25,26,27,28,31,32].

Accordingly, this paper targets motion continuity by combining a control-oriented shared-return model with stage-dependent objective reconstruction and constrained receding-horizon execution.

The main contributions are summarized as follows:

(1) A sensor-interpretable shared-return disturbance-propagation model is developed for networked electro-hydraulic actuators. The model connects neighboring-valve action, common return-line pressure variation, T-port replenishment, accumulator buffering, net driving pressure-difference fluctuation, and local velocity response, thereby explaining how motion and pressure sensor signals reflect the shared-return disturbance path.
(2) A sensor-informed constrained predictive-control formulation is established for motion-continuity preservation. Local displacement, velocity, and pressure-related feedback variables are combined with known neighboring-branch action information over the prediction horizon. Displacement tracking, return-line pressure suppression, and valve-command smoothness are coordinated under a hard valve-command amplitude constraint, a soft return-pressure constraint, and control-increment regularization.
(3) A feasibility-preserving sensor-informed SAC-DMPC strategy is proposed. The SAC policy reconstructs the objective weights of the lower-level DMPC according to the measured motion state, return-line pressure state, neighboring-action information, and recent control behavior. Bounded mapping and smoothing keep the reconstructed weights positive and bounded. Under the stated assumptions, the positive weighting matrices and slack-variable penalty preserve the strict convexity and unique solvability of the lower-level quadratic programming problem, while the soft pressure constraint supports practical feasibility.
(4) The method is validated through coupled mechanical–hydraulic–control simulation, scaled hydraulic-platform experiments, and additional simulation-based ablation and timing tests. The results verify the shared-return disturbance mechanism, the effect of sensor-informed objective-weight reconstruction, the disturbance-attenuation advantage over PID and fixed-weight MPC, and the real-time computational feasibility of the proposed controller.

The remainder of this paper is organized as follows. Section 2 develops the nonlinear dynamic model of the shared-return hydraulic actuator network. Section 3 formulates the locally linearized prediction model and constrained predictive controller. Section 4 presents the feasibility-preserving learning-enhanced predictive-control strategy and analyzes the solvability of the lower-level optimization problem under online objective reconstruction. Section 5 reports the validation setup, SAC training, co-simulation results, simulation-based ablation and baseline comparisons, online computation time, and scaled hydraulic experimental validation. Section 6 concludes the paper.

## 2. Nonlinear Dynamic Modeling of Shared-Return Hydraulic Actuator Networks

A compact nonlinear model is established for a representative actuator unit connected to a shared supply–return hydraulic network. The model includes the dominant pressure–flow–motion coupling mechanisms, including valve nonlinear flow, chamber compressibility, common return-line pressure dynamics, T-port replenishment, accumulator buffering, and neighboring-branch disturbance transmission.

**Figure 1:**
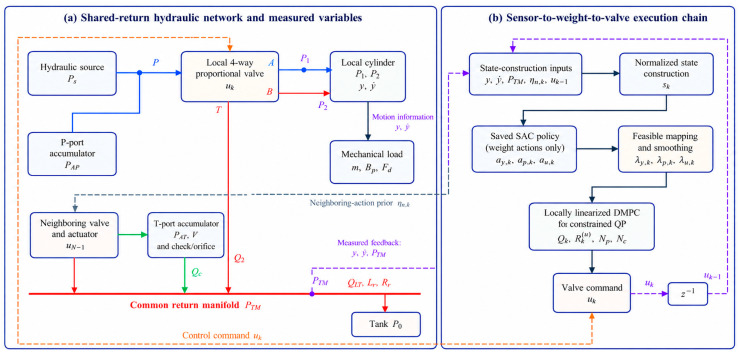
Integrated schematic of (**a**) the shared-return hydraulic network and sensor locations and (**b**) the sensor-informed SAC-DMPC execution chain.

Figure 1 maps the physical shared-return network to the signals used by the controller. The local proportional valve drives the two cylinder chambers, the neighboring branch changes the common return-manifold boundary, and the T-port replenishment and accumulator branches buffer the transient. Displacement and pressure channels, together with the neighboring-action flag, are converted into the upper-level state; the learning layer then reconstructs objective weights, while the lower-level constrained optimizer alone generates the valve command.

### 2.1. Hydraulic Cylinder-Load Nonlinear Dynamics

The local execution unit consists of a valve-controlled hydraulic cylinder and an equivalent mechanical load. This unit is the direct channel through which return-side pressure variation is converted into local motion fluctuation. In the hydraulic-support pushing scenario, it corresponds to a support pushing jack; in a general shared hydraulic network, it represents a valve-controlled actuator affected by both its own control input and the coupled boundary state of the shared return line.

The mechanical dynamics and two-chamber pressure dynamics of the local execution unit are expressed as follows.

(1) $$m\ddot{y}=A_{1}P_{1}-A_{2}P_{2}-B_{p}\dot{y}-F_{d}$$

(2) $$\frac{dP_{1}}{dt}=\frac{\beta_{e}}{V_{10}+A_{1}y}\left[Q_{1}-A_{1}\dot{y}-C_{tp}(P_{1}-P_{2})\right]$$

(3) $$\frac{dP_{2}}{dt}=\frac{\beta_{e}}{V_{20}-A_{2}y}[C_{tp}(P_{1}-P_{2})+A_{2}\dot{y}-Q_{2}]$$

In Equations (1)–(3), m denotes the equivalent moving mass; y, $\dot{y}$, and $\ddot{y}$ denote the cylinder displacement, velocity, and acceleration, respectively; $A_{1}$ and $A_{2}$ are the effective piston areas of the cap-side and rod-side chambers, respectively; $P_{1}$ and $P_{2}$ are the corresponding chamber pressures; $B_{p}$ is the equivalent viscous damping coefficient; $F_{d}$ is the equivalent external load; $\beta_{e}$ is the effective fluid bulk modulus; $V_{10}$ and $V_{20}$ are the initial chamber volumes; $Q_{1}$ is the cap-side inflow; $Q_{2}$ is the rod-side discharge flow; and $C_{tp}$ is the internal leakage coefficient between the two chambers.

Equations (1)–(3) describe the balance of hydraulic force, external load, damping, inertia, chamber flow, leakage, and fluid compressibility. Friction-model selection can materially affect cylinder-load prediction [33]; here, unresolved mechanical effects are represented by the equivalent damping and load terms to retain a compact control-oriented model. The common return-line pressure enters the rod-side discharge boundary and the net driving pressure difference, thereby coupling neighboring return-side transients to local velocity. This cylinder-load model provides the motion-response basis for the shared-line analysis and controller design.

### 2.2. Return-Line Coupling Dynamics and Adjacent-Support Disturbance Analysis

In a shared return network, the local actuator is influenced not only by its own valve opening and load condition, but also by neighboring-unit actions transmitted through the common return manifold. When a neighboring branch starts, stops, or changes valve opening, the local flow balance in the shared hydraulic network changes. The resulting return-line pressure transient modifies the discharge boundary of the controlled actuator and can generate velocity fluctuation even without a large change in the local valve command.

To describe this propagation process, the neighboring T-port replenishment branch and its pressure-difference-driven conduction logic are introduced. The replenishment flow, common return-node pressure, and manifold flow are modeled in a unified manner as follows [34].

(4) $$\sigma_{c}=\left\{\begin{array}{ll} 1, & P_{AT}^{\left(N-1\right)}>P_{TM}\\ 0, & P_{AT}^{\left(N-1\right)}\leq P_{TM} \end{array}\right.$$

(5) $$Q_{c}=\sigma_{c}C_{d}A_{c}\sqrt{\frac{2\left(P_{AT}^{\left(N-1\right)}-P_{TM}\right)}{\rho }}$$

(6) $$\frac{dP_{AT}^{\left(N-1\right)}}{dt}=-\frac{nP_{AT}^{\left(N-1\right)}}{V_{gT}}Q_{c}$$

(7) $$\frac{dP_{TM}}{dt}=\frac{\beta_{m}}{V_{p}}\left(Q_{2}+Q_{c}-Q_{LT}\right)$$

(8) $$P_{TM}-P_{0}=L_{r}\frac{dQ_{LT}}{dt}+R_{r}Q_{LT}$$

In Equations (4)–(8), $\sigma_{c}$ denotes the binary conduction state of the neighboring T-port replenishment branch; $P_{AT}^{\left(N-1\right)}$ is the accumulator-side pressure of the neighboring T-port branch; $P_{TM}$ is the common return-manifold pressure; $Q_{c}$ is the replenishment flow; $C_{d}$ and $A_{c}$ are the discharge coefficient and effective opening area, respectively; ρ is the fluid density; n is the gas polytropic exponent; $V_{gT}$ is the gas volume of the T-port accumulator; $\beta_{m}$ and $V_{p}$ are the effective bulk modulus and control volume of the return manifold, respectively; $Q_{LT}$ is the downstream return flow; $P_{0}$ is the tank pressure; and $L_{r}$ and $R_{r}$ are the inertance and resistance of the return line, respectively.

Equations (4)–(8) describe the nonlinear coupling among neighboring actuator action, the T-port replenishment branch, and the common return manifold. The neighboring actuator action first changes the local flow distribution in the shared hydraulic network. The resulting pressure variation propagates through the common return manifold and changes the discharge boundary of the local actuator. Once the pressure difference across the T-port replenishment branch satisfies the conduction condition, the accumulator-side branch supplies compensating flow to the common return node, thereby attenuating return-side pressure fluctuation and improving the local pressure-retention capability.

This process introduces a piecewise nonlinear mechanism into the shared return-line dynamics. Similar piecewise hydraulic boundary variation caused by relief flow, valve opening, and nonlinear damping has been shown to produce complex transient responses in hydraulic valve and damper systems [34,35]. When the replenishment branch is inactive, the common return manifold is mainly governed by the flow exchange among the local branch, the neighboring branch, and the downstream return path. When the replenishment branch is activated, the accumulator provides transient flow compensation and changes the pressure recovery trajectory of the return node. Consequently, the local velocity response is not simply proportional to the return-line pressure fluctuation. Instead, it is jointly determined by the shared return-line impedance, the neighboring-unit flow disturbance, the conduction state of the replenishment branch, and the local cylinder-load dynamics.

For subsequent predictive-control implementation, the conduction state of the replenishment branch is frozen within each control interval. This treatment preserves the dominant switching nonlinearity of the T-port replenishment process while enabling local linearization and online optimization in the lower-level predictive controller.

### 2.3. Compact Nonlinear State-Space Representation

After the local actuator equations and shared return-line coupling relations are established, the system is arranged into a compact nonlinear state-space representation. This representation clarifies the dynamic connection among local displacement, velocity, chamber pressures, common return-manifold pressure and flow, and the neighboring T-port replenishment branch. It also provides the plant model required for local linearization and prediction-model construction.

The compact nonlinear model is expressed as follows.

(9) $$\dot{X}=f\left(X,u,d\right)$$

The state vector is defined in Equation (10):

(10) $$X=\left[\begin{array}{l} x_{1}\\ x_{2}\\ x_{3}\\ x_{4}\\ x_{5}\\ x_{6}\\ x_{7}\\ x_{8}\\ x_{9} \end{array}\right]=\left[\begin{array}{l} y\\ \dot{y}\\ P_{1}\\ P_{2}\\ P_{AP}\\ Q_{LP}\\ P_{TM}\\ Q_{LT}\\ P_{AT}^{\left(N-1\right)} \end{array}\right]$$

The resulting state equations are given in Equation (11).

(11) $$\begin{array}{l} {\dot{x}}_{1}=x_{2}\\ {\dot{x}}_{2}=\frac{A_{1}x_{3}-A_{2}x_{4}-B_{p}x_{2}-F_{d}}{m}\\ {\dot{x}}_{3}=\frac{\beta_{e}}{V_{10}+A_{1}x_{1}}\left[\begin{array}{l} C_{d}wK_{u}u\sqrt{\frac{2\left(x_{5}-x_{3}\right)}{\rho }}\\ -A_{1}x_{2}-C_{tp}\left(x_{3}-x_{4}\right) \end{array}\right]\\ {\dot{x}}_{4}=\frac{\beta_{e}}{V_{20}-A_{2}x_{1}}\left[\begin{array}{l} C_{tp}\left(x_{3}-x_{4}\right)+A_{2}x_{2}\\ -C_{d}wK_{u}u\sqrt{\frac{2\left(x_{4}-x_{7}\right)}{\rho }} \end{array}\right]\\ {\dot{x}}_{5}=\frac{nx_{5}}{V_{gP}}\left[x_{6}-C_{d}wK_{u}u\sqrt{\frac{2\left(x_{5}-x_{3}\right)}{\rho }}\right]\\ {\dot{x}}_{6}=\frac{P_{s}-x_{5}-R_{s}x_{6}}{L_{s}}\\ {\dot{x}}_{7}=\frac{\beta_{m}}{V_{p}}\left[\begin{array}{l} C_{d}wK_{u}u\sqrt{\frac{2\left(x_{4}-x_{7}\right)}{\rho }}\\ +Q_{c}\left(x_{7},x_{9}\right)-x_{8} \end{array}\right]\\ {\dot{x}}_{8}=\frac{x_{7}-P_{0}-R_{r}x_{8}}{L_{r}}\\ {\dot{x}}_{9}=-\frac{nx_{9}}{V_{gT}}Q_{c}\left(x_{7},x_{9}\right) \end{array}$$

In Equations (9)–(13), X denotes the nonlinear state vector; u is the local normalized valve-control input; and d denotes the neighboring-branch disturbance or prior input. The states $x_{1}$–$x_{9}$ correspond to the cylinder displacement y, cylinder velocity $\dot{y}$, cap-side pressure $P_{1}$, rod-side pressure $P_{2}$, P-port accumulator pressure $P_{AP}$, supply-line flow $Q_{LP}$, common return-manifold pressure $P_{TM}$, return-line flow $Q_{LT}$, and neighboring T-port accumulator pressure $P_{AT}^{\left(N-1\right)}$, respectively. The function f denotes the nonlinear vector field; $P_{s}$ is the supply pressure; $C_{d}$, w, and $K_{u}$ are the valve discharge coefficient, valve-area gradient, and command gain, respectively; $V_{gP}$ is the gas volume of the P-port accumulator; $L_{s}$ and $R_{s}$ are the supply-line inertance and resistance, respectively; $y_{o}$ is the selected output vector; and C is the output-selection matrix.

In this state-space representation, the state variables include the local displacement and velocity, cylinder pressures, shared return-manifold pressure and flow, and the neighboring-unit replenishment-branch states. Therefore, the model explicitly covers the main dynamic variables through which shared return-line disturbance affects the local actuation response. The neighboring-unit action enters the model as an external disturbance or known prior input, while the T-port replenishment branch introduces a state-dependent switching effect. As a result, the system can be regarded as a compact piecewise nonlinear dynamic system with shared hydraulic-network coupling.

Since the coordinated-control objective concerns both local motion continuity and shared-line pressure stability, the local displacement and shared return-manifold pressure are selected as system outputs in Equations (12) and (13).

(12) $$y_{o}=\left[\begin{array}{c} y\\ P_{TM} \end{array}\right]=\left[\begin{array}{c} x_{1}\\ x_{7} \end{array}\right]=CX$$

(13) $$C=\left[\begin{array}{ccccccccc} 1 & 0 & 0 & 0 & 0 & 0 & 0 & 0 & 0\\ 0 & 0 & 0 & 0 & 0 & 0 & 1 & 0 & 0 \end{array}\right]$$

The selected outputs reflect two key aspects of the control problem. The local displacement represents the motion-tracking requirement of the actuator, whereas the shared return-manifold pressure reflects the intensity of return-side hydraulic disturbance. In addition, the net driving pressure difference obtained from the chamber-pressure states provides an important physical indicator for interpreting velocity fluctuation and pressure-retention capability under neighboring-unit disturbance.

Through the above modeling, a nonlinear state-space model is obtained for mechanism analysis, local linearization, prediction-model construction, and controller embedding. Compared with a single-actuator model with a constant return boundary, the proposed model explicitly includes neighboring-unit disturbance propagation, shared return-line impedance, T-port replenishment, and accumulator buffering. It therefore captures the essential nonlinear dynamic mechanism by which shared return-line coupling affects local motion continuity.

### 2.4. Model Characteristics and Role in Subsequent Control Design

The established nonlinear model has three characteristics that are important for the subsequent control design.

First, the model is nonlinear. The nonlinearities originate from valve pressure–flow relations, oil compressibility, chamber-volume variation, pressure-difference-driven replenishment, and the switching logic of the T-port branch. These nonlinear factors jointly determine the transient response of the local actuator under neighboring-unit disturbance.

Second, the model is network-coupled. The local actuator is not only driven by its own valve command and mechanical load, but also affected by neighboring-unit actions through the shared return manifold. This coupling explains why the local velocity and net driving pressure difference may fluctuate even when the local actuator maintains a relatively smooth control input.

Third, the model is suitable for predictive-control embedding. By freezing the conduction state of the replenishment branch within each sampling interval and locally linearizing the nonlinear model around the current operating point, a linear time-varying prediction model can be constructed for online constrained optimization. Therefore, the model serves as the bridge between nonlinear disturbance-propagation analysis and the learning-enhanced predictive controller developed in the following sections.

## 3. Locally Linearized Prediction Model and Constrained Predictive Control

Based on the nonlinear model in Section 2, this section constructs a locally linearized prediction model and a constrained predictive controller. The term distributed refers to controller deployment across actuator units and to the use of neighboring-unit information in local optimization. The derivation is presented for a representative local unit, while neighboring actions are incorporated as known prior inputs when available.

### 3.1. Local Linearization and Discrete Prediction Model

Because the shared-return plant contains valve-flow, chamber-volume, pressure–flow, and T-port switching nonlinearities, a single fixed linear model is inadequate across steady and transition stages. The nonlinear model is therefore linearized about the current operating point at each sampling instant, following the general rationale of efficient local linearization for hydraulically actuated multibody systems [36], and then discretized for online prediction.

The continuous-time system is written in Equations (14) and (15).

(14) $$\dot{X}\left(t\right)=f\left(X\left(t\right),u\left(t\right),w\left(t\right)\right)$$

(15) $$y_{o}\left(t\right)=CX\left(t\right)$$

(16) $$\delta \dot{X}\left(t\right)=A_{k}\delta X\left(t\right)+B_{k}\delta u\left(t\right)+D_{k}\delta w\left(t\right)$$

(17) $$\delta y_{o}\left(t\right)=C\delta X\left(t\right)$$

The corresponding deviation model after local linearization is given by Equations (16) and (17).

To improve numerical stability in the presence of the high bulk modulus of the hydraulic system, the bilinear transform (Tustin method) is employed to discretize the local linear model. With the controller sampling period denoted by $T_{s}$, the corresponding discrete-time model is obtained as Equations (18) and (19).

(18) $$\delta X_{k+1}=A_{d,k}\delta X_{k}+B_{d,k}\delta u_{k}+D_{d,k}\delta w_{k}$$

(19) $$\delta y_{o,k}=C\delta X_{k}$$

In Equations (14)–(19), w(t) denotes the known neighboring-action or disturbance input used in the prediction model; δ denotes the deviation from the current operating point; $A_{k}$, $B_{k}$, and $D_{k}$ are the continuous-time Jacobian matrices with respect to the state, valve-control input, and neighboring input, respectively; $A_{d,k}$, $B_{d,k}$, and $D_{d,k}$ are the corresponding matrices obtained through Tustin discretization; k is the sampling index; and $T_{s}$ is the controller sampling period. These matrices are recalculated at each sampling instant based on the current local operating point before constructing the prediction model.

In the present study, the conveyor-pushing displacement and the pressure of the common return manifold are selected as the controlled outputs for subsequent prediction-model construction and optimization.

### 3.2. Compact Prediction Model Based on Control Increments

Let the prediction horizon and the control horizon be $N_{p}$ and $N_{c}$, respectively, with $N_{c}\leq N_{p}$. After defining the control-increment sequence and the predicted sequence of known disturbances, the future state and output trajectories over the prediction horizon can be recursively derived from the discrete-time state equation. Since the control objectives are directly formulated in the output space, an output prediction model based on control increments is further established, as shown in Equation (20).

(20) $$Y_{k}=\Phi_{k}\delta X_{k}+\Theta_{k}\Delta U_{k}+\Gamma_{k}W_{k}+\Xi_{k}u_{k-1}$$

(21) $$\Theta_{k}=H_{k}L,\Xi_{k}=H_{k}1$$

In Equations (20)–(25), $Y_{k}$ denotes the stacked predicted-output vector; $\Delta U_{k}$ is the future control-increment sequence; $\Phi_{k}$, $\Theta_{k}$, $\Lambda_{k}$, and $\Xi_{k}$ are the lifted free-response, control-response, known-disturbance-response, and previous-input-response matrices, respectively; $E_{k}$ collects the current deviation and known offset terms; $R_{k}$ is the reference sequence; $Q_{k}$ and $R_{k}^{\left(u\right)}$ are the output-error and control-increment weighting matrices, respectively; $\rho_{\varepsilon }$ is the slack-variable penalty coefficient; $\varepsilon_{k}$ is the non-negative pressure-constraint slack variable; $H_{k}$ and $g_{k}$ are the Hessian matrix and gradient vector of the quadratic programming problem; $G_{k}$ and $b_{k}$ are the inequality-constraint matrix and bound vector; $Z_{k}$ is the decision vector; $N_{p}$ and $N_{c}$ are the prediction horizon and control horizon, respectively; and $\Delta u_{k|k}^{*}$ is the first optimized control increment applied to $u_{k}$.

The prediction model in Equation (20) consists of the free response caused by the current state deviation, the forced response induced by future control increments, the accumulated influence of neighboring-unit prior information, and the offset related to the previous control input. Therefore, the neighboring-unit action is not treated merely as an unknown random disturbance. When neighboring information is available, its influence on the shared return line can be incorporated into the prediction horizon, which provides the basis for predictive pre-adjustment of the valve command before the disturbance is fully reflected in the local velocity response.

### 3.3. Constrained Receding-Horizon Optimization Formulation

At each sampling instant, a finite-horizon constrained optimization problem is solved in a receding-horizon manner to coordinate local motion tracking, shared return-line pressure regulation, and valve-command smoothness. The general formulation accommodates constraints on the valve-command amplitude, control increment, and return-manifold pressure. In the implementation used for validation, the active hard constraint is imposed on the cumulative valve command as 0≤u≤1. The control increment is retained as the optimization variable and penalized through the positive weight $r_{u}$, while no independent hard increment-rate constraint is enforced.

(22) $$J_{k}={\left(Y_{k}-R_{k}\right)}^{T}Q_{k}(Y_{k}-R_{k})+\Delta U_{k}^{T}R_{k}^{\left(u\right)}\Delta U_{k}+\rho_{\varepsilon }\varepsilon_{k}^{2}$$

By substituting the prediction model into the objective function, the optimization problem can be reformulated into the standard quadratic form, whose Hessian matrix and gradient vector are given in Equation (23).

(23) $$\begin{array}{l} H_{k}=\left[\begin{array}{cc} 2\left(\Theta_{k}^{T}Q_{k}\Theta_{k}+R_{k}^{\left(u\right)}\right) & 0\\ 0 & 2\rho_{\varepsilon } \end{array}\right]\\ g_{k}=\left[\begin{array}{c} 2\Theta_{k}^{T}Q_{k}E_{k}\\ 0 \end{array}\right] \end{array}$$

After the active implementation constraints are assembled, the optimization is solved in the standard quadratic programming form of Equation (24). The return-pressure upper bound is 35 MPa and is softened by a non-negative slack variable with a penalty coefficient of $10^{4}$, whereas the valve-command amplitude bound remains hard. This combination prevents a temporary pressure excursion from making the lower-level QP infeasible while preserving bounded valve execution.

(24) $$\begin{array}{c} \underset{Z_{k}}{\min}\frac{1}{2}Z_{k}^{T}H_{k}Z_{k}+g_{k}^{T}Z_{k}\\ s.t.G_{k}Z_{k}\leq b_{k}\\ \varepsilon_{k}\geq 0 \end{array}$$

After solving this problem, only the first element of the optimal control-increment sequence is applied to the system, as shown in Equation (25). The controller then proceeds to the next sampling instant and repeats the receding-horizon procedure of state update, local linearization, rolling optimization, and first-move implementation.

(25) $$u_{k}=u_{k-1}+\Delta u_{k|k}^{*}$$

For the reported validation, the lower-level DMPC was implemented with the sampling period $T_{s}=0.05\;s$, the prediction and control horizons $N_{p}=N_{c}=5$, bounded adaptive weights, Tustin discretization, and the MATLAB quadprog solver. At each sampling instant, only the first element of the optimized control-increment sequence $\Delta U_{k}$ was applied to update the valve command. 

## 4. Feasibility-Preserving Learning-Enhanced Predictive Control

Although the constrained predictive controller in Section 3 provides an interpretable optimization framework, fixed objective weights are not sufficient for the sensor-observed stage-dependent dynamics of a shared-return hydraulic network. During steady advancing, displacement tracking and input smoothness should dominate. This motivates an upper-level learning module that uses sensor-derived feedback variables to adapt the objective weights online while leaving the constrained valve-command computation to the lower-level DMPC.

### 4.1. Markov Decision Modeling of the Upper-Level Weight-Adaptation Module

The controller state is constructed through a sensor-to-state-to-objective chain. The cylinder displacement y is obtained from the displacement channel, while the velocity-related information is provided by the synchronized motion channel or calculated from sampled displacement by the acquisition and state-processing layer. The common return-manifold pressure $P_{TM}$ is used to obtain the return-pressure deviation and its rate. The neighboring-action prior $\eta_{n,k}$ is constructed from time-stamped adjacent valve-command or action-state messages transmitted through the support-controller network. The present validation assumes synchronization with the local sampling instant. Communication delay shortens the available preview time, whereas packet loss interrupts the neighboring-prior update; the local displacement, velocity, and return-pressure feedback remain available. For practical deployment, short communication gaps can be handled by retaining the latest valid message, whereas persistent communication loss can be handled by setting $\eta_{n,k}=0$ and switching to conservative fixed weights.

After normalization, the displacement error, velocity error, return-pressure deviation, their corresponding rates, the neighboring-action prior, and the previous valve input constitute the state vector $s_{k}$ in Equation (26). The SAC policy maps $s_{k}$ to three bounded weight-adjustment actions, which reconstruct the weighting matrices $Q_{k}$ and $R_{k}^{\left(u\right)}$ through feasible-set mapping and smoothing. The lower-level DMPC then computes the valve command $u_{k}$ by solving the constrained quadratic programming problem.

(26) $$s_{k}=\left[\begin{array}{l} {\tilde{e}}_{y,k}\\ {\dot{\tilde{e}}}_{y,k}\\ {\tilde{e}}_{p,k}\\ {\dot{\tilde{e}}}_{p,k}\\ \eta_{n,k}\\ {\tilde{u}}_{k-1} \end{array}\right]$$

The action of the upper-level agent is defined by Equation (27) as a three-dimensional continuous vector bounded in $\left[-1,1\right]$, corresponding to the adjustment commands for the displacement-tracking weight, the pressure-suppression weight, and the control-increment weight, respectively.

(27) $$a_{k}={\left[\begin{array}{ccc} a_{y,k} & a_{p,k} & a_{u,k} \end{array}\right]}^{T}\in {\left(-1,1\right)}^{3}$$

To ensure that the lower-level DMPC weights always remain within a prescribed feasible range, the agent outputs are mapped to weight commands through an affine transformation, as shown in Equation (28). To avoid numerical oscillation in the lower-level optimizer caused by abrupt weight changes, the actual weights applied to the DMPC are further updated through a first-order smoothing law, as shown in Equation (29). Accordingly, the output-weighting matrix and the input-weighting matrix in Section 3 are reconstructed online according to Equation (30).

(28) $$\begin{array}{l} \lambda_{y,k}^{\mathrm{cmd}}=\lambda_{y}^{\min}+\frac{a_{y,k}+1}{2}\left(\lambda_{y}^{\max}-\lambda_{y}^{\min}\right)\\ \lambda_{p,k}^{\mathrm{cmd}}=\lambda_{p}^{\min}+\frac{a_{p,k}+1}{2}\left(\lambda_{p}^{\max}-\lambda_{p}^{\min}\right)\\ \lambda_{u,k}^{\mathrm{cmd}}=\lambda_{u}^{\min}+\frac{a_{u,k}+1}{2}\left(\lambda_{u}^{\max}-\lambda_{u}^{\min}\right) \end{array}$$

(29) $$\begin{array}{l} \lambda_{y,k}=\left(1-\beta_{\lambda }\right)\lambda_{y,k-1}+\beta_{\lambda }\lambda_{y,k}^{\mathrm{cmd}}\\ \lambda_{p,k}=\left(1-\beta_{\lambda }\right)\lambda_{p,k-1}+\beta_{\lambda }\lambda_{p,k}^{\mathrm{cmd}}\\ \lambda_{u,k}=\left(1-\beta_{\lambda }\right)\lambda_{u,k-1}+\beta_{\lambda }\lambda_{u,k}^{\mathrm{cmd}} \end{array}$$

(30) $$Q_{k}=I_{N_{p}}⊗\left[\begin{array}{cc} \lambda_{y,k} & 0\\ 0 & \lambda_{p,k} \end{array}\right],R_{k}=\lambda_{u,k}I_{N_{c}}$$

To guide the agent toward a proper trade-off among displacement-tracking accuracy, pressure-disturbance suppression, and control smoothness, the immediate reward is designed in the form of Equation (31), where the safety penalty term is defined by Equation (32).

(31) $$r_{k}=-c_{y}{\tilde{e}}_{y,k}^{2}-c_{p}{\tilde{e}}_{p,k}^{2}-c_{d}{\dot{\tilde{e}}}_{y,k}^{2}-c_{u}{\left(\frac{\Delta u_{k}}{\Delta u_{\max}}\right)}^{2}-c_{a}\|a_{k}-a_{k-1}{\|}_{2}^{2}-p_{\mathrm{safe},k}$$

(32) $$\begin{array}{l} p_{\mathrm{safe},k}=k_{p}\left[\max{\left(0,P_{TM,k}-P_{TM}^{\max}\right)}^{2}+\max{\left(0,P_{TM}^{\min}-P_{TM,k}\right)}^{2}\right]\frac{1}{P_{\mathrm{sc}}^{2}}+\\ k_{y}\left[\max{\left(0,y_{k}-y^{\max}\right)}^{2}+\max{\left(0,y^{\min}-y_{k}\right)}^{2}\right]\frac{1}{y_{\mathrm{sc}}^{2}} \end{array}$$

In Equations (26)–(34), $s_{k}$ denotes the normalized SAC state vector; $e_{y,k}$ and ${\dot{e}}_{y,k}$ are the displacement-tracking error and its rate; $e_{p,k}$ and ${\dot{e}}_{p,k}$ are the return-pressure deviation and its rate; $\eta_{n,k}$ is the neighboring-action prior; and $u_{k-1}$ is the valve command at the previous sampling instant. The superscript ~ denotes normalized variables. The action components $a_{y,k}$, $a_{p,k}$, and $a_{u,k}$ adjust the displacement-tracking, pressure-suppression, and input-smoothness weights, respectively. The coefficients $c_{y}$, $c_{p}$, $c_{d}$, $c_{u}$, and $c_{a}$ are the weighting coefficients associated with displacement tracking, pressure deviation, error-rate variation, control increment, and action variation, respectively; $\Delta u_{\max}$ is the normalization bound of the control increment; $p_{\mathrm{safe},k}$ is the soft safety-penalty term; $k_{p}$ and $k_{y}$ are the pressure- and displacement-penalty gains; $P_{\mathrm{sc}}$ and $y_{\mathrm{sc}}$ are the corresponding scaling factors; and the superscripts min and max denote the prescribed operating bounds. In Equation (33), $\mu_{\phi }$ and $\sigma_{\phi }$ are the mean and standard-deviation outputs of the actor network; $\xi_{k}$ is a standard Gaussian random variable; and the hyperbolic tangent function bounds the sampled action within the prescribed interval.

The SAC module performs state-dependent objective reallocation among displacement tracking, return-pressure regulation, and control-input smoothness. The bounded mapping and first-order smoothing update maintain all applied weights within the prescribed feasible set, and the learned policy determines the direction and magnitude of each adjustment from the current sensor-derived state. The reconstructed weights are passed to the lower-level DMPC for valve-command optimization.

### 4.2. SAC Network Architecture and Offline Training Strategy

The SAC algorithm is adopted to learn the upper-level weight-adjustment policy because it is suitable for continuous action spaces and provides stable exploration through entropy regularization. The actor network produces the continuous weight-adjustment action, while two critic networks estimate soft state-action values to reduce overestimation. The actor network generates bounded objective-weight adjustments, and the lower-level DMPC computes the hydraulic valve command.

(33) $${\tilde{a}}_{k}=\mu_{\phi }(s_{k})+\sigma_{\phi }(s_{k})⊙\epsilon_{k},\epsilon_{k}\sim N(0,I)$$

(34) $$a_{k}=\tanh({\tilde{a}}_{k})$$

During offline training, the replay-buffer capacity and mini-batch size are set to $1\times 10^{6}$ transitions and 256, respectively. The entropy-temperature coefficient is adjusted automatically, with an initial value of 1 and a learning rate of $3\times 10^{-4}$, while the target entropy is determined automatically. The target networks are updated at each learning step using a soft-update coefficient of 0.005. During co-simulation and scaled hydraulic-platform validation, the policy-network parameters remain fixed, and the reported results are obtained using one saved SAC policy checkpoint.

### 4.3. Hierarchical Closed-Loop Execution of SAC-Based Weight Adaptation and Predictive Control

The hierarchical closed-loop execution is therefore as follows. At each sampling instant, the sensor-derived state vector is sent to the trained SAC policy, which outputs a weight-adjustment action. After bounded mapping and smoothing, the reconstructed weights are passed to the lower-level DMPC. The nonlinear hydraulic model is then locally linearized, the constrained QP is solved, and only the first valve-command increment is applied. This receding-horizon procedure is repeated at the next sampling instant.

### 4.4. Feasibility Preservation and Solvability Under Online Weight Adaptation

The SAC module reconstructs the objective weights, and the constrained QP generates the valve command. The affine weight mapping and first-order smoothing restrict the applied weights to positive and bounded intervals, thereby preserving the numerical properties of the lower-level optimizer during online adaptation.

#### 4.4.1. Weight Feasible-Set Constraints and Online Safe Mapping

According to the continuous action output of the SAC agent in Section 4.1 and the weighting structure of the DMPC objective function in Section 3, the feasible set of the objective weights is defined by Equations (35) and (36), in which all weights are restricted to positive and bounded intervals.

(35) $$\Omega_{\lambda }=\left\{{\left[\begin{array}{ccc} \lambda_{y} & \lambda_{p} & \lambda_{u} \end{array}\right]}^{T}\;|\;\lambda_{y}\in [\lambda_{y,\min},\lambda_{y,\max}],\lambda_{p}\in [\lambda_{p,\min},\lambda_{p,\max}],\lambda_{u}\in [\lambda_{u,\min},\lambda_{u,\max}]\right\}$$

(36) $$\begin{array}{l} 0<\lambda_{y,\min}<\lambda_{y,\max}\\ 0<\lambda_{p,\min}<\lambda_{p,\max}\\ 0<\lambda_{u,\min}<\lambda_{u,\max} \end{array}$$

The upper-level action is given by Equation (37), and the corresponding weight commands are generated through the affine mapping in Equation (38).

(37) $$a_{k}={\left[a_{y,k},a_{p,k},a_{u,k}\right]}^{T}\in {\left[-1,1\right]}^{3}$$

(38) $$\begin{array}{l} \lambda_{y,k}^{\mathrm{cmd}}=\lambda_{y,\min}+\frac{a_{y,k}+1}{2}\left(\lambda_{y,\max}-\lambda_{y,\min}\right)\\ \lambda_{p,k}^{\mathrm{cmd}}=\lambda_{p,\min}+\frac{a_{p,k}+1}{2}\left(\lambda_{p,\max}-\lambda_{p,\min}\right)\\ \lambda_{u,k}^{\mathrm{cmd}}=\lambda_{u,\min}+\frac{a_{u,k}+1}{2}\left(\lambda_{u,\max}-\lambda_{u,\min}\right) \end{array}$$

To suppress numerical oscillation in the lower-level optimizer caused by abrupt weight variation, the actual weights delivered to the DMPC are further updated through the first-order smoothing law in Equation (39). The output-weighting matrix and the input-weighting matrix at the k-th sampling instant are then reconstructed according to Equation (40).

(39) $$\begin{array}{l} \lambda_{y,k}=(1-\beta_{\lambda })\lambda_{y,k-1}+\beta_{\lambda }\lambda_{y,k}^{\mathrm{cmd}}\\ \lambda_{p,k}=(1-\beta_{\lambda })\lambda_{p,k-1}+\beta_{\lambda }\lambda_{p,k}^{\mathrm{cmd}}\\ \lambda_{u,k}=(1-\beta_{\lambda })\lambda_{u,k-1}+\beta_{\lambda }\lambda_{u,k}^{\mathrm{cmd}} \end{array}$$

(40) $$\begin{array}{l} Q_{k}=I_{N_{p}}⊗\left[\begin{array}{cc} \lambda_{y,k} & 0\\ 0 & \lambda_{p,k} \end{array}\right]\\ R_{k}^{\left(u\right)}=\lambda_{u,k}I_{N_{c}} \end{array}$$

From the boundedness of the action, the affine mapping, and the first-order smoothing update, it follows that, if the initial weights lie within the prescribed feasible set, the updated weights at all subsequent instants remain within the same set, as expressed in Equation (41).

(41) $${\left[\begin{array}{ccc} \lambda_{y,k} & \lambda_{p,k} & \lambda_{u,k} \end{array}\right]}^{T}\in \Omega_{\lambda }$$

This indicates that the proposed mapping and smoothing mechanism renders the weight feasible set positively invariant during online adaptation.

#### 4.4.2. Solvability of the Lower-Level Optimization and Closed-Loop Boundedness

Under the weight-feasible-set constraints, the lower-level DMPC optimization problem at the k-th sampling instant can still be written in the standard quadratic programming form shown in Equations (42)–(44).

(42) $$\begin{array}{l} \min\frac{1}{2}Z_{k}^{T}H_{k}Z_{k}+g_{k}^{T}Z_{k}\\ s.t.\;\;G_{k}Z_{k}\leq b_{k}\\ \varepsilon_{k}\geq 0 \end{array}$$

(43) $$Z_{k}={\left[\Delta U_{k}^{T},\varepsilon_{k}\right]}^{T}$$

(44) $$H_{k}=\left[\begin{array}{cc} 2\left(\Theta_{k}^{T}Q_{k}\Theta_{k}+R_{k}^{\left(u\right)}\right) & 0\\ 0 & 2\rho_{\varepsilon } \end{array}\right]$$

In Equations (35)–(44), $\Omega_{\lambda }$ denotes the compact feasible set of strictly positive objective weights; the superscripts min, max, and cmd denote the lower bounds, upper bounds, and mapped weight commands, respectively; $\beta_{\lambda }$ is the first-order weight-smoothing factor; $I_{N_{p}}$ and $I_{N_{c}}$ are identity matrices associated with the prediction and control horizons; $Q_{k}$ and $R_{k}^{\left(u\right)}$ are the reconstructed block weighting matrices; $Z_{k}$ contains the control-increment sequence and the pressure-constraint slack variable; $\rho_{\varepsilon }$ is the positive slack-variable penalty coefficient; and $H_{k}$, $g_{k}$, $G_{k}$, and $b_{k}$ are the Hessian matrix, gradient vector, inequality-constraint matrix, and inequality-bound vector of the lower-level quadratic programming problem, respectively. Under the stated local-model and feasibility assumptions, the strictly positive bounded weights together with $\rho_{\varepsilon }>0$ preserve the positive definiteness of $H_{k}$.

The reconstructed output- and input-increment weighting matrices remain positive definite and bounded, and the slack-variable penalty remains positive. Accordingly, the Hessian matrix of the lower-level quadratic program remains positive definite, the objective function is strictly convex, and the optimizer is unique whenever the constraint set is nonempty.

The non-negative slack variable represents temporary return-pressure excursions caused by disturbances or model mismatch and retains feasibility with respect to the softened pressure constraint. In the reported implementation, the hard constraint $0\leq u_{k}\leq 1$ limits the cumulative valve command, while the control increment is regularized by the positive weighting matrix $R_{k}^{\left(u\right)}$ without imposing an additional hard increment-rate constraint. Under bounded objective weights, bounded disturbances, bounded linearization residuals, feasible hard-input constraints, local stabilizability, recursive feasibility of the lower-level quadratic programming problem, and a local decrease condition of the receding-horizon value function, the applied valve command remains bounded, and the displacement-tracking error and return-line pressure deviation converge to a bounded residual set within the considered operating region. Under these conditions, the hierarchical controller preserves the feasible weight set and the strict convexity of the lower-level optimization problem, while the closed-loop response remains locally practically bounded.

The following result characterizes preservation of the weight feasible set, solvability of the lower-level quadratic programming problem, and local practical boundedness of the closed-loop response within the considered operating region under the stated assumptions [28,32,37,38,39].

**Proposition** **1.** *Local feasibility preservation and practical boundedness under online weight adaptation.*

Suppose that the weight feasible set is compact and strictly positive, the initial weights belong to this feasible set, the external disturbance and local linearization residual are bounded, and the locally linearized prediction model is stabilizable within the considered operating region. Assume further that the lower-level quadratic programming problem is recursively feasible and that the corresponding receding-horizon value function satisfies a local decrease condition. Then, the proposed hierarchical SAC-DMPC controller preserves the positive definiteness of the weighting matrices during online weight adaptation. The lower-level quadratic programming problem remains strictly convex and admits a unique optimizer at each sampling instant. Under the hard input constraint and soft return-pressure constraint, the valve command remains bounded, and the displacement-tracking error and return-line pressure deviation remain locally practically bounded.

**Proof.**  Let the feasible weight set be compact and convex, and let the smoothing factor satisfy $0\leq \beta_{\lambda }\leq 1$. The bounded affine mapping generates weight commands within the prescribed feasible set, while the first-order smoothing update forms a convex combination of the previous feasible weights and the current mapped commands. Therefore, feasible initial weights imply that all subsequent weights remain within the prescribed set. Since the weight lower bounds are strictly positive, the reconstructed output- and control-increment-weighting matrices remain positive definite and uniformly bounded.

Under the stated local-model and feasibility assumptions, the positive weighting matrices together with the positive slack-variable penalty preserve the positive definiteness of the Hessian matrix $H_{k}$. Consequently, the lower-level quadratic programming problem remains strictly convex and admits a unique optimizer whenever its constraint set is nonempty. The hard valve-amplitude constraint bounds the applied command, while the positive control-increment weight penalizes abrupt command variation. No independent hard increment-rate constraint is imposed in the reported implementation.

The return-line pressure constraint is softened by the non-negative slack variable $\varepsilon_{k}$. Hence, temporary pressure excursions caused by bounded disturbances or model mismatch can be accommodated by the slack variable and do not independently render the optimization problem infeasible. Under bounded disturbances, bounded linearization residuals, local stabilizability, recursive feasibility, and the standard local decrease condition of the receding-horizon value function, the displacement-tracking error and return-line pressure deviation converge to a bounded residual set. Therefore, the closed-loop response remains locally practically bounded within the considered operating region. □

The bounded weight mapping, first-order smoothing update, and soft pressure constraint preserve the feasible weight set, lower-level QP solvability, and bounded valve execution during online objective reconstruction.

## 5. Validation and Discussion

To evaluate disturbance attenuation, constrained-control behavior, and implementability, this section presents results from coupled co-simulation, simulation-based ablation and baseline tests, online timing evaluation, and scaled hydraulic-platform experiments. All reported validation data were obtained from completed simulation or experimental runs: Section 5.3 uses ADAMS-AMESim-Simulink co-simulation, Section 5.4, Section 5.5 and Section 5.6 use MATLAB simulation-based controller validation with the trained SAC agent and lower-level DMPC model, and Section 5.7 uses the scaled dual-branch hydraulic experimental platform. The validation is organized around three questions: whether neighboring-branch actions generate measurable shared-return disturbances in the pressure and motion signals, whether sensor-informed SAC-DMPC reduces the conversion of hydraulic disturbance into velocity and pressure-difference fluctuations, and whether the added learning layer remains computationally feasible for online control.

### 5.1. Validation Setup and Disturbance Scenario

The neighboring disturbance is generated by commanded start–stop or valve-state transitions of an adjacent branch. In the co-simulation, the neighboring-branch transitions occur at *t* = 4.0 s and *t* = 6.0 s, while the coupled branch switches at *t* = 5.0 s in the scaled-platform experiment. These events correspond to adjacent-support action transitions during sequential advancing or conveyor-pushing coordination. The disturbance magnitude is jointly determined by the neighboring-valve opening, branch flow demand, accumulator state, and shared return-line impedance. The selected cases characterize switching-induced shared-return disturbances under the considered operating conditions. The validation covers the specified load level, valve-switching rate, network configuration, and disturbance timings.

The coupled validation environment consists of a mechanical layer, a hydraulic layer, and a control layer. The mechanical layer is implemented in ADAMS to describe the motion, contact, and load transmission between the hydraulic supports and the scraper conveyor. The hydraulic layer is implemented in AMESim to describe the hydraulic dynamics of the supply pipeline, common return manifold, valve-controlled cylinder, P-port accumulator, and T-port replenishment branch. The control layer is implemented in MATLAB/Simulink to perform state processing, SAC-based weight inference, DMPC receding-horizon optimization, and valve-command input/output. Through variable exchange among the three software environments, mechanical loads, hydraulic pressure–flow states, and control commands are updated cooperatively within the same closed loop, as summarized in Table 1.

**Figure 2:**
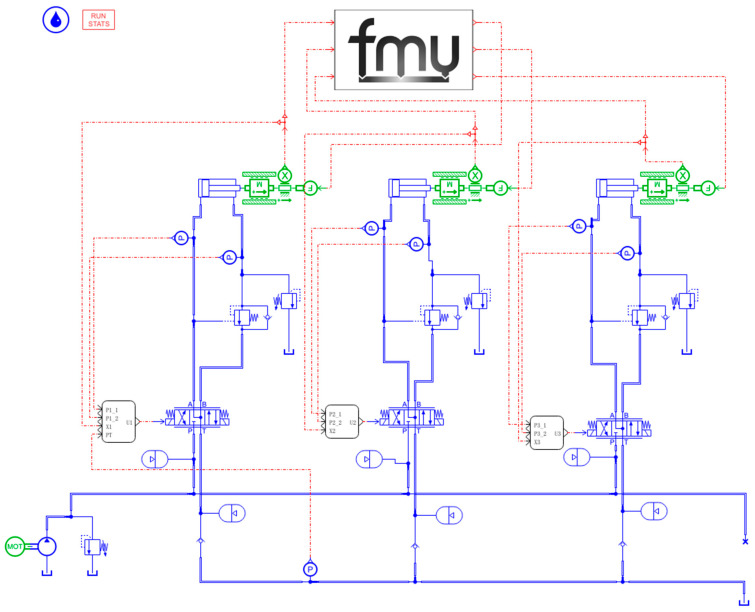
Coupled mechanical–hydraulic–control validation framework for shared-return disturbance analysis.

In this framework, ADAMS provides displacement, velocity, and mechanical-side load information to the hydraulic and control layers. AMESim feeds back cylinder pressures, common return-line pressure, flow states, and valve-controlled execution states to the control layer. Simulink generates the objective weights and solves the predictive optimization problem according to the real-time states, and then sends the valve command back to the AMESim hydraulic model. In this way, a closed-loop coupled validation environment is established for shared return-line disturbance analysis and constrained controller verification, as illustrated in Figure 2.

The co-simulation and scaled-platform experiment address different aspects of the validation. The co-simulation is used to analyze shared return-line disturbance propagation, T-port replenishment and accumulator buffering, and the closed-loop response under mechanical–hydraulic–control coupling. The scaled-platform experiment evaluates controller implementation using physical hydraulic components, sensor feedback, proportional-valve actuation, and neighboring-branch disturbance generation. The two validation environments provide mechanism-level analysis and implementation-oriented evidence under the considered operating conditions.

The SAC policy is trained offline, and the learned policy parameters remain fixed during closed-loop validation. The validation stage evaluates the nonlinear system response, constrained DMPC execution, and valve-command generation under coupled electro-hydraulic and mechanical dynamics.

### 5.2. SAC Offline Training and Online Weight-Inference Behavior

The SAC policy used in this study was obtained from one completed offline training run without a fixed MATLAB random seed. The single retained checkpoint was used throughout the subsequent co-simulation, ablation, timing, and scaled-platform tests. During validation, the policy parameters remained fixed and only forward inference was performed.

**Figure 3:**
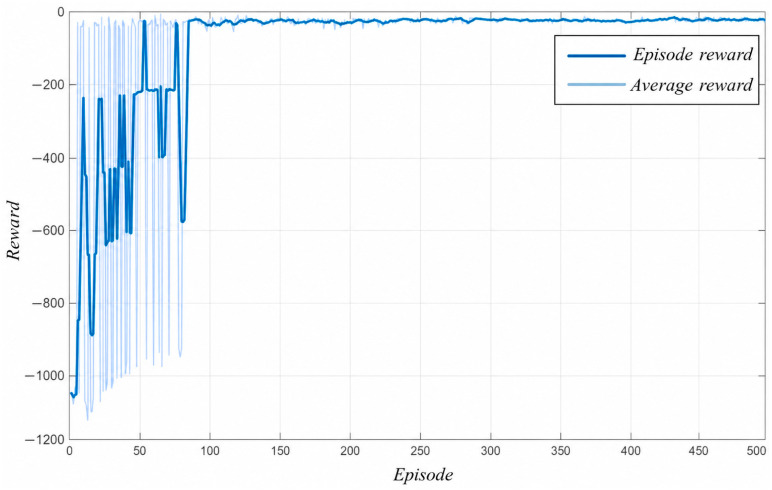
Reward evolution of the SAC agent during offline training.

The training environment employed the shared-return disturbance mechanism described above, with the disturbance applied over t∈[3.4,4.0] s. The subsequent co-simulation used neighboring-branch transitions at *t* = 4.0 s and *t* = 6.0 s, whereas the scaled-platform test used a switching event at *t* = 5.0 s. Training and validation used independent state trajectories, and the validation trajectories and experimental records were excluded from gradient updates. The episode reward and average reward during offline SAC training are shown in Figure 3.

During closed-loop validation, the parameters of the trained SAC policy remain fixed, and only forward inference is performed. At each sampling instant, the normalized state vector $s_{k}$, constructed from sensor-derived motion-error information, return-pressure information, the neighboring-action prior, and the previous valve command $u_{k-1}$, is mapped to bounded weight-adjustment actions. After feasible-set mapping and smoothing, these actions update the objective weights of the lower-level DMPC. The lower-level DMPC then solves the constrained quadratic programming problem based on the locally linearized prediction model and applies the first optimized control increment to update the valve command $u_{k}$. The main offline SAC training, lower-level DMPC implementation, and computational-environment parameters are summarized in Table 2.

In the lower-level DMPC implementation, the prediction and control horizons are set to $N_{p}=N_{c}=5$, and only the first optimized control increment is applied at each sampling instant. The hard constraint $0\leq u_{k}\leq 1$ limits the cumulative valve command, while the control increment is penalized through the positive weighting matrix $R_{k}^{\left(u\right)}$. No independent hard increment-rate constraint is imposed. The Tustin method was used for local prediction-model discretization, whereas the nonlinear plant states were propagated using a fourth-order Runge–Kutta scheme with 10 integration substeps within each 0.05 s control interval, corresponding to an integration step of 0.005 s. Training robustness across independently seeded runs was not assessed. No separate held-out set of plant parameters, load conditions, or disturbance amplitudes was used; the training–validation separation was based on independent state trajectories and different disturbance timings.

### 5.3. Co-Simulation Results Under Neighboring Disturbances

**Figure 4:**
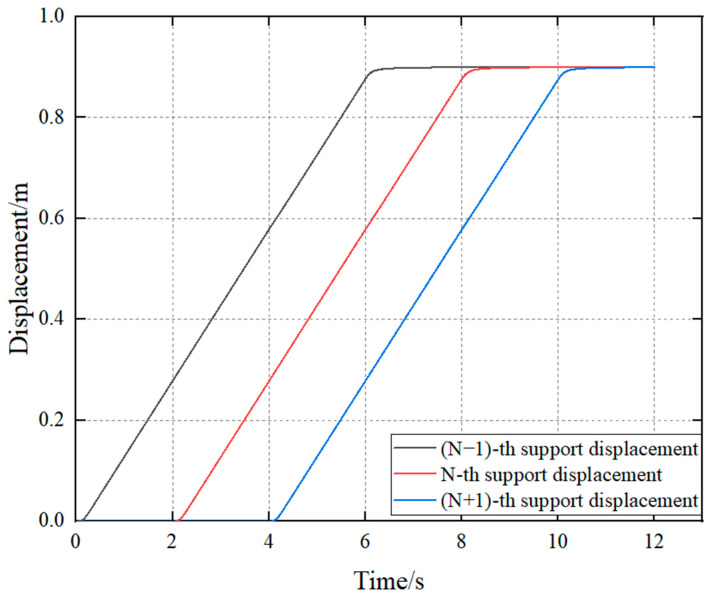
Displacement responses of the three supports.

**Figure 5:**
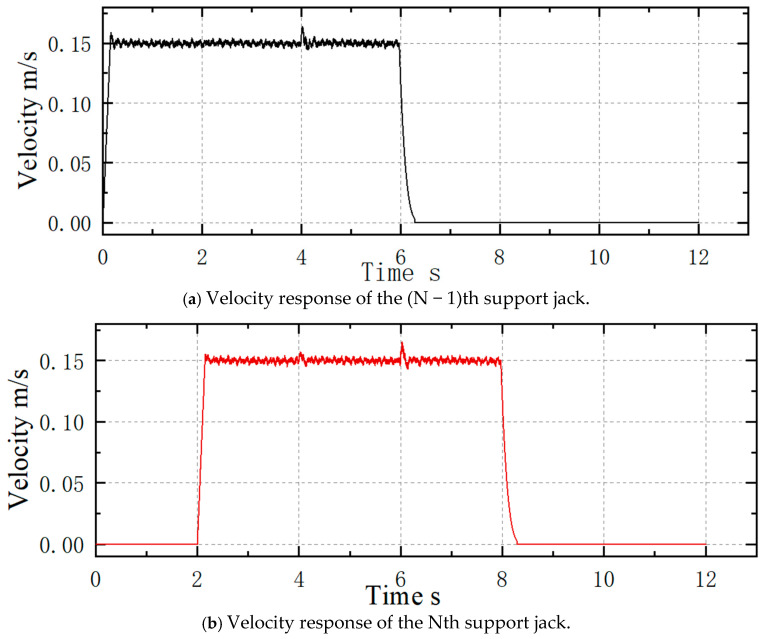

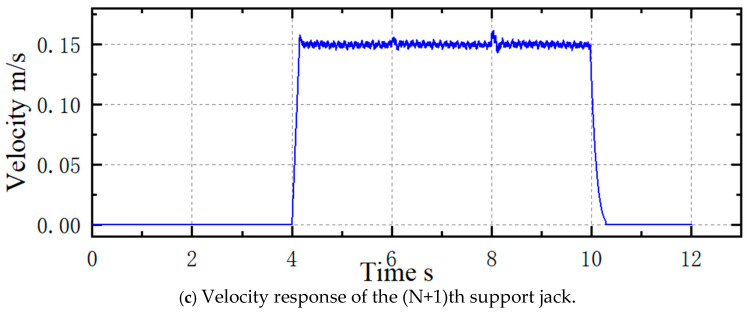
Velocity responses of the three support jacks during coordinated advancing.

Figure 4 and Figure 5 show the coordinated displacement and velocity responses of the three supports. The displacement trajectories complete the prescribed advancing sequence, whereas short velocity transients occur near neighboring-branch transitions. These paired responses establish the motion-side signature of shared-return coupling before the pressure and valve signals are examined.

**Figure 6:**
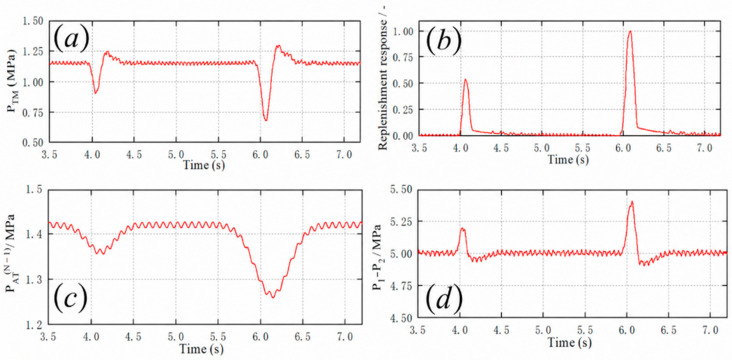
Coupled hydraulic responses under shared return-line disturbance: (**a**) common return-manifold pressure; (**b**) T-port replenishment-branch response; (**c**) neighboring T-port accumulator pressure; (**d**) local cylinder chamber pressure difference.

The motion responses are consistent with the modeled propagation path: neighboring actions perturb the common return boundary, while the T-port branch and accumulator reshape the transient before it appears in local velocity. Figure 6 examines this hydraulic-side sequence.

Figure 6 reports the common return-manifold pressure, replenishment response, accumulator pressure, and chamber pressure difference around the two neighboring-branch transitions.

After each neighboring transition, the return-manifold pressure falls, the replenishment branch activates, and accumulator pressure decreases as stored fluid is released. The chamber pressure difference rises temporarily, with a larger response at the second transition. Together with Figure 5, these signals support the modeled direction of propagation from the shared return boundary to local motion under the tested condition.

**Figure 7:**
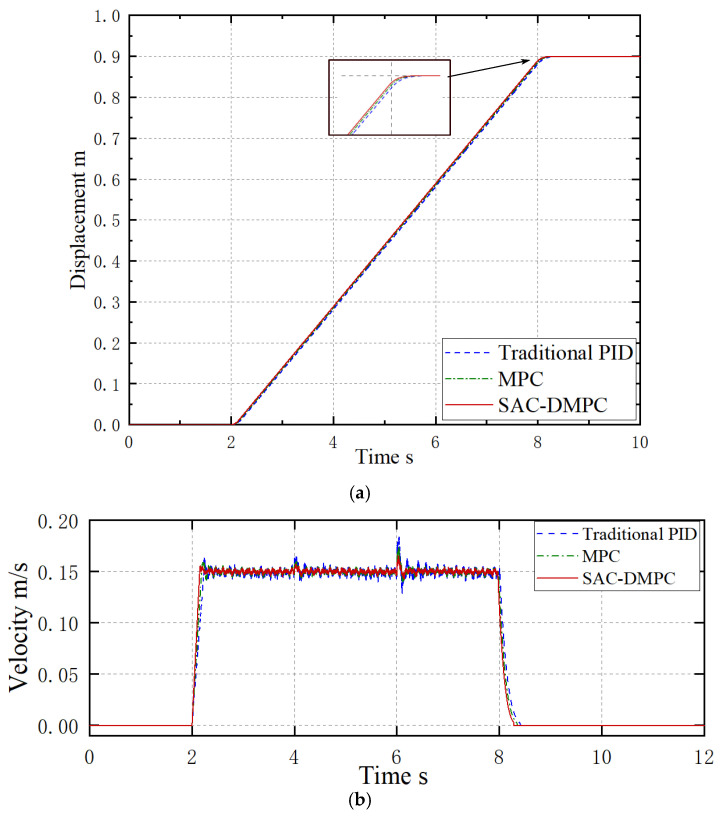
Controller comparison for the Nth support: (**a**) displacement responses; (**b**) velocity responses.

To evaluate controller performance under shared return-line disturbance, the N-th support is selected as the representative unit, and SAC-DMPC is compared with PID and fixed-weight MPC. Figure 7 shows that all controllers can drive the actuator to the target displacement, but their disturbance-recovery behavior differs clearly. PID reacts mainly after velocity deviation has occurred. Fixed-weight MPC improves prediction and constraint handling, but its objective preference remains unchanged across steady and disturbance stages. SAC-DMPC shows faster target arrival, smaller velocity fluctuation, and better recovery after both neighboring disturbances.

Table 3 quantitatively confirms these observations. SAC-DMPC achieves the shortest target-displacement arrival time of 8.134 s. Under the two neighboring disturbances, its velocity overshoots are 0.0071 m/s and 0.0156 m/s, respectively, both of which are lower than those obtained with PID and fixed-weight MPC.

These differences arise from objective allocation rather than a change in the lower-level constraints. PID responds after local error appears, and fixed-weight MPC retains one objective balance. SAC-DMPC uses the measured state and neighboring prior to alter that balance before and during each transition.

**Figure 8:**
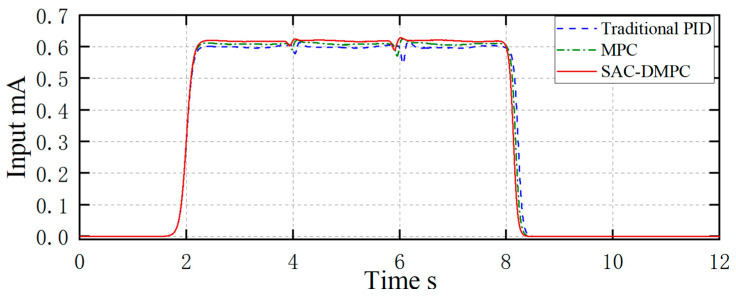
Valve-command responses of PID, fixed-weight MPC, and SAC-DMPC.

Figure 8 compares the valve commands. The predictive controllers adjust earlier than PID, while SAC-DMPC adds small state-dependent corrections without losing overall input continuity.

The control curves indicate that the tracking improvement is not obtained through a large increase in valve-command variation.

**Figure 9:**
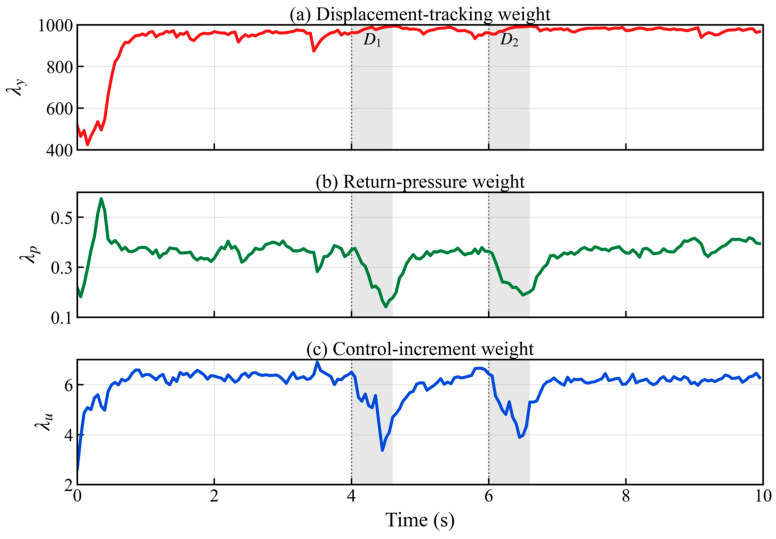
Mapped and smoothed DMPC objective weights following neighboring-branch transitions at 4.0 s and 6.0 s.

Figure 9 reports the mapped and smoothed objective weights from the two-event controller-level validation. The dashed vertical lines mark neighboring-branch transitions at 4.0 s and 6.0 s; the shaded regions highlight the subsequent response intervals for visual comparison rather than the duration of the switching commands. Separate panels are used because the three weights have different numerical ranges (Table 2).

Figure 9 confirms that the mapped weights remain within the prescribed bounds and vary smoothly after the transitions at 4.0 s and 6.0 s. In both cases, the tracking-related weight changes moderately, whereas the return-pressure and control-increment weights decrease temporarily and then recover. Because these weights have different scales and multiply different error terms, their absolute magnitudes should not be interpreted as direct measures of control priority. The repeated response supports state-dependent objective reconstruction while the soft pressure constraint remains active [22,31,32].

### 5.4. Simulation-Based Ablation Study

To further verify the contribution of the proposed modules, additional simulation-based ablation tests were completed in MATLAB using the trained SAC agent and the lower-level constrained DMPC validation model. The same controller structure and sampling setting were used, while one module was removed or fixed at a time. This validation complements the co-simulation and hydraulic-platform experiments by isolating the function of neighboring prior information, adaptive pressure weighting, weight smoothing, and the soft pressure constraint.

**Figure 10:**
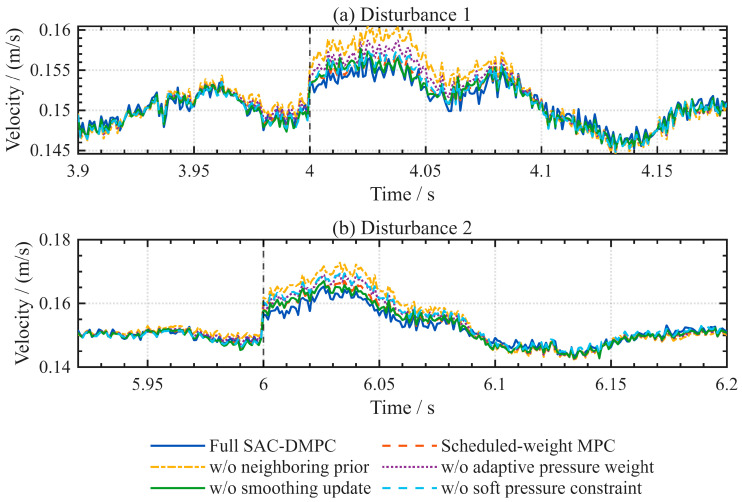
Simulation-based ablation velocity responses of SAC-DMPC variants.

The ablation results reveal trade-offs rather than uniform dominance. Removing the neighboring prior or smoothing increases overshoot; fixing the pressure weight gives zero measured overshoot, and removing the soft pressure constraint gives a slightly smaller overshoot than the full controller. The full configuration retains adaptive pressure weighting and soft constraint handling while achieving moderate arrival time and input variation, with no QP failure or hard-constraint violation. Thus, Table 4 isolates the role of each module across tracking, motion continuity, input variation, and feasibility. The corresponding velocity responses of the SAC-DMPC ablation variants are shown in Figure 10.

### 5.5. Stronger Baseline Comparison

The scheduled-weight MPC baseline uses the same constrained MPC layer as SAC-DMPC and applies a manually specified stage-dependent weight schedule. It provides a rule-based adaptive-weight reference for evaluating the state-dependent objective reconstruction generated by the SAC policy.

Table 5 shows the same multi-objective trade-off. Fixed-weight MPC minimizes overshoot but has the longest arrival time and greater input variation than SAC-DMPC. Scheduled-weight MPC reaches the target fastest but yields the largest overshoot and input variation. SAC-DMPC provides intermediate arrival and overshoot values and the smallest total input variation, with no QP failure or constraint violation in the tested run. It is therefore interpreted as a constrained compromise, not as the best controller for every scalar metric.

### 5.6. Online Computational Time

The online computation time was measured by instrumenting SAC inference, local model linearization, QP construction, QP solution, and the total control update in the MATLAB simulation-validation environment. MATLAB warm-up was completed before timing, and the first 10 samples were excluded from the reported stable-segment statistics to remove model-loading and just-in-time compilation overhead. The component-wise online computation times of the full SAC-DMPC controller are summarized in Table 6.

The full controller required 4.289 ms on average and 6.899 ms at maximum over the reported stable segment, both below the 50.0 ms sampling period. No QP failure or hard-constraint violation occurred, supporting sampled execution for this implementation.

### 5.7. Scaled Hydraulic Experimental Validation

**Figure 11:**
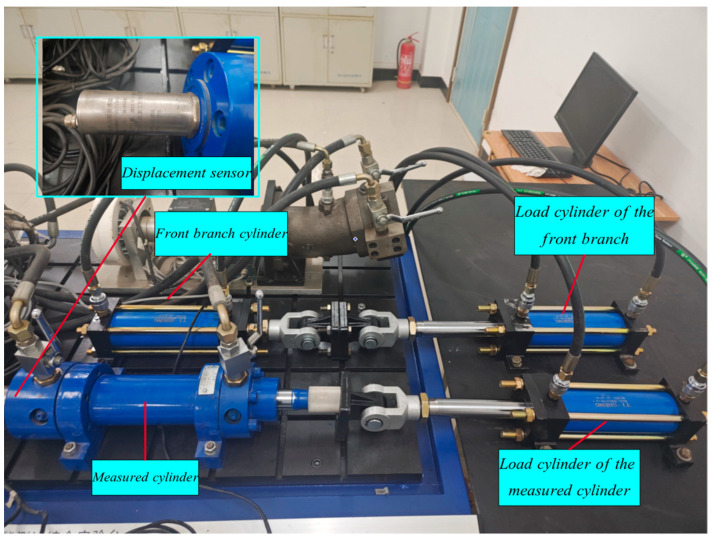
Main actuating cylinder experimental platform.

**Figure 12:**
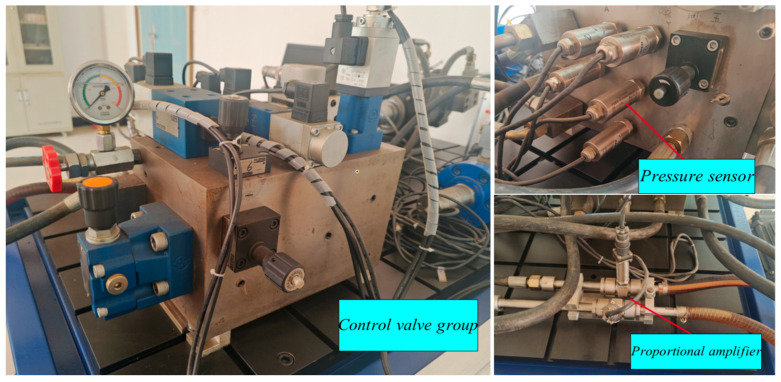
Control valve group.

**Figure 13:**
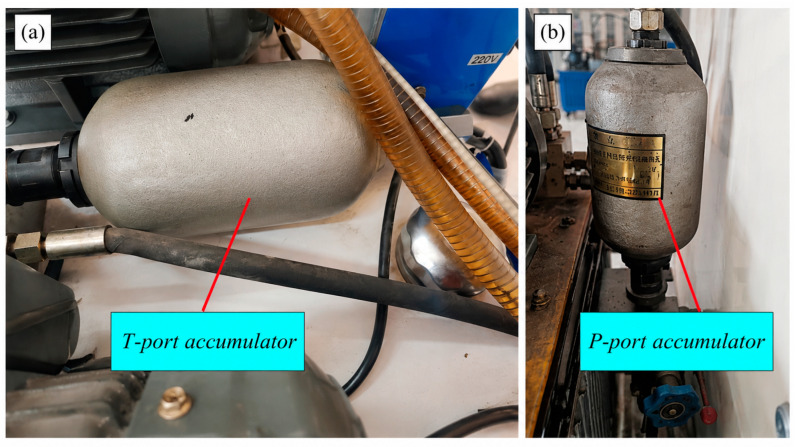
Local view of measured cylinder.

To verify implementability in an actual hydraulic circuit, a dual-branch scaled experimental platform with a shared supply–return line is constructed. The platform includes a hydraulic source, active cylinder, opposed load cylinder, electro-hydraulic proportional valve group, displacement and pressure sensors, data-acquisition module, and control system. Neighboring-branch disturbance is generated through the motion variation in the coupled branch, which emulates the shared-line disturbance path considered in the model. Figure 11, Figure 12 and Figure 13 show the main actuating-cylinder experimental platform, the control valve group, and a local view of the measured cylinder, respectively, while the main parameters of the scaled experimental platform are summarized in Table 7.

**Figure 14:**
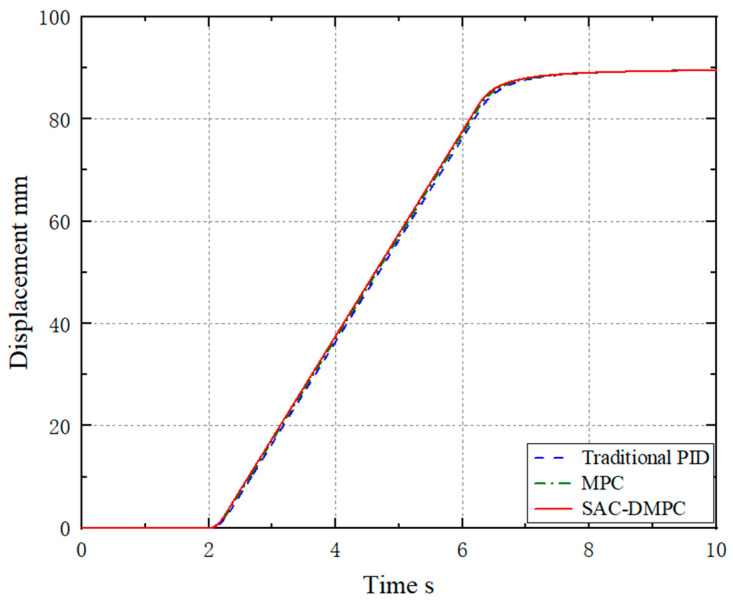
Displacement curves of measured cylinder.

**Figure 15:**
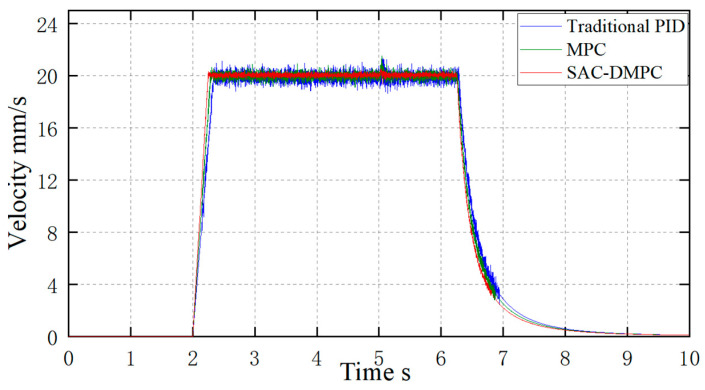
Velocity curves of measured cylinder.

**Figure 16:**
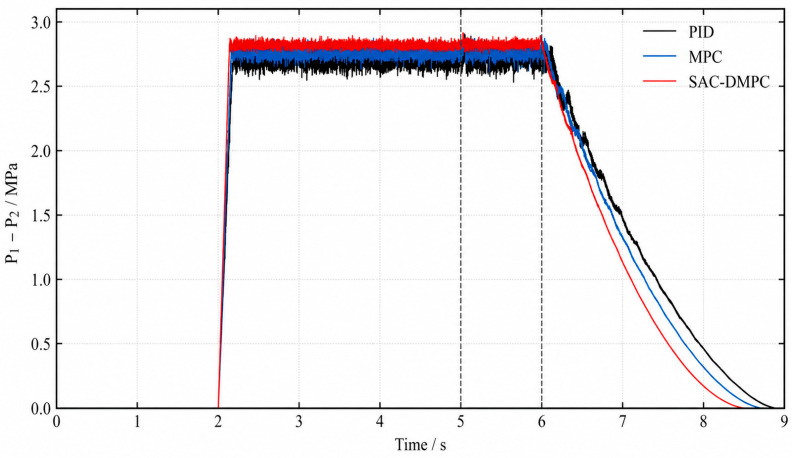
Net driving pressure-difference curves of measured cylinder.

One formal test was conducted for each controller, including PID, fixed-weight MPC, and SAC-DMPC. The complete records from each test are reported in Figure 14, Figure 15 and Figure 16; therefore, no selection among repeated runs was involved. All tests were conducted with a nominal source pressure of 10 MPa, a target displacement of 90 mm, identical normalized valve-command limits, and a neighboring-branch switching time of *t* = 5.0 s. The archived signals were sampled at 0.001 s, and the velocity indices were calculated directly from the recorded velocity channel without additional numerical differentiation or smoothing. Displacement was measured using the built-in displacement sensor of the measured cylinder, while chamber pressures were acquired from the pressure sensors integrated into the valve group. Detailed metrological and calibration information was unavailable; therefore, sensor-level uncertainty intervals are not reported. 

Figure 14 and Figure 15 present the displacement and velocity responses of the measured cylinder under the scaled-platform validation conditions. The velocity indices in Table 8 are calculated from the same processed records and are uniformly expressed in mm/s without additional smoothing. The mean pre-disturbance velocity is calculated over t∈[4.0,5.0) s, and the post-disturbance peak velocity is identified within t∈[5.0,5.4] s. The velocity overshoot is defined as the difference between the post-disturbance peak velocity and the mean pre-disturbance velocity.

As shown in Figure 14 and Figure 15 and summarized in Table 8, SAC-DMPC yielded a post-disturbance peak velocity of 20.5570 mm/s and a velocity overshoot of 0.5136 mm/s, corresponding to an overshoot percentage of 2.5627%. Within the specified evaluation window, the velocity overshoot was 65.1% and 66.4% lower than those of PID and fixed-weight MPC, respectively. These results indicate a smaller disturbance-induced velocity increment under the tested operating condition.

In addition to displacement and velocity, the net driving pressure difference is used to characterize the hydraulic-side disturbance increment. Figure 16 and Table 9 are derived from the same processed pressure-difference dataset. The mean pre-disturbance pressure difference is calculated over t∈[4.0,5.0) s, and the post-disturbance peak is identified within t∈[5.0,5.4] s. The pressure-difference overshoot is defined as the difference between the post-disturbance peak and the mean pre-disturbance value.

As shown in Figure 16 and Table 9, SAC-DMPC yielded a net driving pressure-difference overshoot of 0.0980 MPa, corresponding to an overshoot percentage of 3.4780%. Within the specified evaluation window, the overshoot was 52.6% and 19.4% lower than those of PID and fixed-weight MPC, respectively. The absolute post-disturbance peak pressure difference was 2.9164 MPa, indicating that the improvement was mainly reflected in the disturbance-induced increment relative to the pre-disturbance operating level.

For practical deployment, the displacement and pressure channels can be monitored through scheduled zero/span checks, range- and rate-plausibility tests, slow-bias estimation, and pressure–motion consistency evaluation. Persistent signal inconsistency can activate a sensor-health flag and switch the controller to conservative fixed weights or a local safe-control mode.

Taken together, the scaled-platform results provide implementation evidence for the physical execution of the fixed SAC policy and constrained DMPC under the reported individual test conditions.

## 6. Conclusions

This study investigated motion-continuity control of shared-return electro-hydraulic actuator networks subject to neighboring-branch disturbances. Neighboring-valve-state transitions change the hydraulic boundary of the common return line, affect the local discharge condition and net driving pressure difference, and subsequently produce velocity fluctuations in the controlled actuator. A compact nonlinear model was established by incorporating supply-line impedance, two-chamber cylinder dynamics, P-port accumulator buffering, common return-line dynamics, and neighboring T-port replenishment.

Based on the locally linearized prediction model, a hierarchical SAC-DMPC controller was developed. The upper-level SAC policy reconstructs the objective weights according to sensor-derived motion and pressure states, neighboring-action prior information, and recent control behavior. The lower-level DMPC solves the constrained quadratic programming problem and generates the valve command. Under the stated assumptions, bounded weight mapping and smoothing preserve the feasible weight set, while the positive weighting matrices and slack-variable penalty maintain the strict convexity and numerical solvability of the lower-level optimization problem.

In co-simulation, SAC-DMPC reached the target in 8.134 s and limited velocity overshoot to 0.0071 and 0.0156 m/s at the two neighboring-branch transitions. These results show that objective reconstruction redistributes tracking, pressure, and input-smoothness penalties across the tested stages.

The dual-branch scaled-platform experiment evaluated controller execution under the tested shared-return disturbance sequence. Within the specified evaluation windows, SAC-DMPC produced a velocity overshoot of 0.5136 mm/s and a net driving pressure-difference overshoot of 0.0980 MPa. Both values were lower than those obtained with PID and fixed-weight MPC. The experimental results indicate a reduction in the disturbance-induced velocity and pressure-difference increments under the considered operating condition.

The ablation and baseline comparisons show that the controller modules affect arrival time, velocity overshoot, total input variation, and constraint handling in different ways. SAC-DMPC yielded a mean online update time of 4.289 ms under a sampling period of 50.0 ms, with no QP failures or hard-constraint violations in the reported run. The SAC results were obtained from one training run, and the physical results characterize the reported individual tests. Future work will quantify multi-seed variability, repeated-test statistics, and sensor-level calibration uncertainty and will extend validation to larger actuator networks, broader load spectra, longer pipeline propagation, communication uncertainty, and sensor-drift compensation.

## Figures and Tables

**Table 1:** Main information exchange relationships in the coupled validation environment.

Validation Layer	Output Variables	Receiver	Function
ADAMS mechanical layer	Displacement, velocity, mechanical load	AMESim/Simulink	Mechanical-state and load feedback
AMESim hydraulic layer	Chamber pressures, common return-line pressure, flow states, valve states	Simulink	Hydraulic-state feedback
MATLAB/Simulink control layer	SAC weights, DMPC control increment, valve command	AMESim	Online control execution
SAC module	Weights related to displacement, pressure, and input	DMPC module	Online objective-function reconstruction
DMPC module	Optimal control increment and valve input	AMESim hydraulic layer	Actuation of valve-controlled unit

**Table 2:** Main SAC training, DMPC implementation, and computational-environment parameters.

Parameter	Value
Controller sampling period, *T_s_*	0.05 s
Training episodes/steps per episode	500/200
Replay-buffer capacity/mini-batch size	1 × 10^6^/256
Actor/critic learning rates	1 × 10^−4^/3 × 10^−4^
Discount factor, *γ*	0.99
Target-network update	Every learning step; *τ* = 0.005
Entropy-temperature adjustment	Automatic; initial coefficient 1; learning rate 3 × 10^−4^
Hidden-layer structure	[256, 256]
SAC action dimension and range	3; each component in [−1, 1]
Prediction and control horizons	*N_p_* = *N_c_* = 5
Valve-command bound	0 ≤ *u_k_* ≤ 1
Soft pressure bound/slack penalty	35 MPa/1 × 10^4^
Adaptive weight ranges	*λ_y_* ∈ [10, 1000]; *λ_p_* ∈ [0.01, 1.0]; *λ_u_* ∈ [0.1, 10]
Weight-smoothing factor	*β_λ_* = 0.30
Prediction-model discretization	Tustin method
QP solver	MATLAB quadprog
Software environment	MATLAB R2025a; Windows 10 ×64
Reinforcement Learning Toolbox	Version 25.1 (R2025a)
Optimization Toolbox	Version 25.1 (R2025a)
Processor	AMD Ryzen 5 5600X
System memory	32 GB
Plant-state integration method	Fourth-order Runge–Kutta
Integration substeps per control interval	10
Plant integration step	0.005 s
Independent SAC training runs	1
MATLAB random seed	Not fixed
Validation checkpoint	Checkpoint retained at training completion after reward stabilization

**Table 3:** Comparison of key performance indicators for the three controllers in co-simulation.

Index	PID	Fixed-Weight MPC	SAC-DMPC
Target-displacement arrival time (s)	8.224	8.174	8.134
Peak time under disturbance 1 (s)	4.038	4.027	4.022
Velocity overshoot under disturbance 1 (m/s)	0.0155	0.0099	0.0071
Peak time under disturbance 2 (s)	6.042	6.033	6.026
Velocity overshoot under disturbance 2 (m/s)	0.0347	0.0253	0.0156

**Table 4:** Simulation-based ablation validation of the SAC-DMPC controller.

Controller Variant	Changed Module	Arrival Time (s)	Velocity Overshoot (m/s)	Input Total Variation (–)	QP Failures (Count)	Constraint Violations (Count)
Full SAC-DMPC	None	0.650	0.012564	1.065	0	0
w/o neighboring prior	Neighboring-branch prior removed	0.600	0.014240	1.073	0	0
w/o adaptive pressure weight	Pressure-related weight fixed	0.650	0.000000	1.071	0	0
w/o smoothing update	Weight smoothing disabled	0.600	0.021491	1.061	0	0
w/o soft pressure constraint	Soft pressure constraint removed	0.600	0.010541	1.057	0	0

**Table 5:** Simulation-based comparison with fixed-weight and scheduled-weight MPC baselines.

Controller	Weight Strategy	Arrival Time (s)	Velocity Overshoot (m/s)	Input Total Variation (–)	QP Failures (Count)	Constraint Violations (Count)
Fixed-weight MPC	Constant manually tuned weights	0.850	0.001089	1.167	0	0
Scheduled-weight MPC	Rule-based staged weights	0.550	0.046225	1.447	0	0
Full SAC-DMPC	SAC-adaptive weights + constrained MPC	0.650	0.012564	1.065	0	0

**Table 6:** Online computation time of the full SAC-DMPC controller.

Component	Mean Time (ms)	Maximum Time (ms)	Standard Deviation (ms)	Note
SAC inference	2.082	4.590	0.488	Upper-level policy evaluation
Linearization	0.057	0.137	0.019	Local hydraulic model update
QP construction	0.273	1.158	0.130	Prediction matrices and constraints
QP solution	1.472	2.357	0.233	Lower-level constrained DMPC solve
Total update	4.289	6.899	0.744	Complete online control update
Sampling period	50.0	–	–	Controller sampling interval
QP failures/constraint violations	0	–	–	0 constraint violations
Timing protocol	warm-up 10	excluded 10	190 samples	Stable online segment after warm-up

**Table 7:** Main parameters of the scaled experimental platform.

Parameter	Value
Rated flow (L/min)	30
Rated pressure (MPa)	10
Working fluid	97% water-based emulsion
Fluid density (kg/m^3^)	1010
Kinematic viscosity (mm^2^/s)	1.5
Bulk modulus (GPa)	0.8
Measured-cylinder bore/rod/stroke (mm)	63/36–150
Target displacement (mm)	90

**Table 8:** Motion-continuity indices calculated from the representative scaled-platform records.

Index	PID	Fixed-Weight MPC	SAC-DMPC
Mean pre-disturbance velocity (mm/s)	19.9084	19.9883	20.0434
Post-disturbance peak velocity (mm/s)	21.3803	21.5180	20.5570
Peak-velocity time (s)	5.069	5.049	5.043
Velocity overshoot (mm/s)	1.4719	1.5297	0.5136
Velocity overshoot percentage (%)	7.3933	7.6530	2.5627

**Table 9:** Net driving pressure-difference indices calculated from the representative scaled-platform records.

Index	PID	Fixed-Weight MPC	SAC-DMPC
Mean pre-disturbance net driving pressure difference (MPa)	2.6981	2.7584	2.8184
Post-disturbance peak net driving pressure difference (MPa)	2.9050	2.8799	2.9164
Peak-pressure-difference time (s)	5.044	5.092	5.027
Net driving pressure-difference overshoot (MPa)	0.2069	0.1216	0.0980
Net driving pressure-difference overshoot percentage (%)	7.6671	4.4069	3.4780

## Data Availability

The data presented in this study are available on request from the corresponding author.
